# Mortality and its predictors in abdominal injury across sub-Saharan Africa: systematic review and meta-analysis

**DOI:** 10.1186/s12873-024-00982-3

**Published:** 2024-04-11

**Authors:** Destaw Endeshaw, Amare Mebrat Delie, Ousman Adal, Abiyu Abadi Tareke, Eyob Ketema Bogale, Tadele Fentabel Anagaw, Misganaw Guadie Tiruneh, Eneyew Talie Fenta

**Affiliations:** 1 Department of Adult Health Nursing, School of Health Science, College of Medicine and Health Science, Bahir Dar University, Bahir Dar, Ethiopia; 2 Department of Public Health, College of medicine and health science, Injibara University, Injibara, Ethiopia; 3 Department of emergency and critical care nursing, School of Health Science, College of Medicine and Health Science, Bahir Dar University, Bahir Dar, Ethiopia; 4 Amref Health in Africa, COVID-19 vaccine/EPI technical assistant at West Gondar zonal health department, Gondar, Ethiopia; 5 Health Promotion and Behavioral science department, College of medicine and health science, Bahir Dar University, Bahir Dar, Ethiopia; 6 Department of Health Systems and Policy, Institute of Public Health, College of Medicine and Health Sciences, University of Gondar, Gondar, Ethiopia

**Keywords:** Abdominal injuries, Meta-analysis, Mortality, Sub-Saharan Africa

## Abstract

**Background:**

Abdominal injuries exert a significant impact on global morbidity and mortality. The aggregation of mortality data and its determinants across different regions holds immense importance for designing informed healthcare strategies. Hence, this study assessed the pooled mortality rate and its predictors across sub-Saharan Africa.

**Method:**

This meta-analysis employed a comprehensive search across multiple electronic databases including PubMed, Africa Index Medicus, Science Direct, and Hinari, complemented by a search of Google Scholar. Subsequently, data were extracted into an Excel format. The compiled dataset was then exported to STATA 17 statistical software for analysis. Utilizing the Dersimonian-Laird method, a random-effect model was employed to estimate the pooled mortality rate and its associated predictors. Heterogeneity was evaluated via the *I*^*2*^ test, while publication bias was assessed using a funnel plot along with Egger's, and Begg's tests.

**Result:**

This meta-analysis, which includes 33 full-text studies, revealed a pooled mortality rate of 9.67% (95% CI; 7.81, 11.52) in patients with abdominal injuries across sub-Saharan Africa with substantial heterogeneity (*I2* = 87.21%). This review also identified significant predictors of mortality. As a result, the presence of shock upon presentation demonstrated 6.19 times (95% CI; 3.70-10.38) higher odds of mortality, followed by ICU admission (AOR: 5.20, 95% CI; 2.38-11.38), blunt abdominal injury (AOR: 8.18, 95% CI; 4.97-13.45), post-operative complications (AOR: 8.17, 95% CI; 4.97-13.44), and the performance of damage control surgery (AOR: 4.62, 95% CI; 1.85-11.52).

**Conclusion:**

Abdominal injury mortality is notably high in sub-Saharan Africa. Shock at presentation, ICU admission, blunt abdominal injury, postoperative complications, and use of damage control surgery predict mortality. Tailored strategies to address these predictors could significantly reduce deaths in the region.

**Supplementary Information:**

The online version contains supplementary material available at 10.1186/s12873-024-00982-3.

## Background

Injuries, whether from accidents or violence, account for around 4.4 million annual fatalities, representing nearly 8% of global deaths. Within this global context, 17.6% of fatalities are attributed to the African region [1]. It is noteworthy that injuries stand as the third leading cause of death globally across all age groups [2]. Moreover, they contribute significantly to about 10% of the global burden of disability [3]. In the context of this larger problem, abdominal injuries whether blunt or penetrating emerge as a crucial component, making a substantial contribution to the overall spectrum of injuries [4, 5]. The abdomen emerges as the third most commonly affected body region, with 7-10% of all trauma-related fatalities attributed to injuries in this area [6]. Traumatic brain injury stands as a predominant factor, contributing to one-third to one-half of all trauma-related fatalities [7]. Following closely, thoracic trauma accounts for approximately 25% of these deaths [8].

Abdominal injuries can have profound and life-threatening consequences for individuals. Their impact spans a range of outcomes, from causing organ damage [9] to severe, life-threatening conditions [4, 10]. The abundance of normal floras within the gastrointestinal system heightens the vulnerability of abdominal injuries to infectious complications [11]. The rupture of major blood vessels within this region also significantly exacerbates the severity of these injuries [12]. Moreover, the abdomen presents a diagnostic challenge often referred to as a "black box," compounding the complexities associated with addressing these injuries [13]. All these factors collectively increase the mortality rates associated with abdominal injuries.

Abdominal injuries cause significant mortality. Globally, a recent systematic review showed a 17% mortality rate from patients presented with blunt abdominal trauma [14]. Despite a lack of comprehensive evidence summaries in Africa, studies have highlighted high mortality rates linked to abdominal injuries in this continent, ranging from 2% [15] to 28% [16]. These rates exhibit considerable variation across diverse geographical settings and periods.

In a prior review, significant risk factors for mortality in patients with abdominal injuries were identified. These factors encompass older age, firearm injuries, associated injuries, vascular injuries, an increased number of red blood cell transfusions, and solid organ injuries [17].

The fragmented state of studies on abdominal injury mortality in sub-Saharan Africa (SSA) underscores the critical need for a comprehensive review and meta-analysis on this issue. Pooled estimates play a vital role in identifying key factors influencing mortality rates, providing essential guidance for clinicians and policymakers. Nevertheless, based on our search, there is currently a lack of synthesized evidence on this topic across sub-Saharan Africa, a region characterized by inadequate healthcare infrastructure and limited resources. Therefore, this review aimed to estimate the pooled mortality rate and its predictors within the SSA region.

## Methods

### Protocol and registration

The findings presented in this review adhere to the guidelines outlined in the Preferred Reporting Items for Systematic Review and Meta-Analysis (PRISMA) statement [18] (Additional file 1). The protocol for this review has been prospectively registered in the International Prospective Register of Systematic Reviews (PROSPERO), under the registration number CRD42023484989.

### Search strategy and selection criteria

To identify relevant studies, we conducted searches across multiple databases including, PubMed, Africa Index Medicus, Science Direct, Hinari, and a search engine, Google Scholar. Our search, carried out from November 10 to 22, 2023, utilized specific keywords such as mortality, predictors, abdominal injuries, and sub-Saharan Africa. Search strategies incorporated various techniques including truncation (*), boolean operators ('OR' and 'AND'), and phrase searching (“...”). Additionally, we employed MeSH terms and synonyms to make our searches comprehensive. The detailed search terms in each database are provided (Additional file 2). Our search was broadened by accessing exclusive digital repositories from Addis Ababa University and Bahir Dar University. A manual search of the included articles' reference lists was also performed to identify additional relevant studies.

### Inclusion and exclusion criteria

This review included diverse studies published in English-language that reported mortality rates and/or predictive factors related to mortality in cases of abdominal injuries, without restricting the study period. To provide additional clarity, the inclusion criteria covered studies that detailed in-hospital mortality and/or the factors contributing to it in patients with abdominal injuries of any type. This inclusion was regardless of whether associated extra-abdominal injuries were present or not, irrespective of the severity status, and regardless of the causative factor. Articles accessible within our search source from November 10-22, 2023, were included. Exclusions comprised articles lacking abstracts or full texts, anonymous reports, editorials, studies lacking clear reporting of outcomes, and qualitative studies.

### Quality assessment and data abstraction procedure

The initial phase involved importing references from the searched databases into EndNote software version 20 to remove duplicates and prepare the references for subsequent processing. Then, two authors (DE and OA) independently reviewed and screened titles and abstracts based on predefined criteria. Following this, full-text articles were retrieved and reviewed independently by both authors. Any discrepancies in selection were resolved through discussion with a third author (EKB). Selected studies underwent a quality assessment for risk of bias using the Joanna Briggs Institute (JBI) critical appraisal checklist tailored for cross-sectional (both descriptive and analytical) and cohort studies. The checklist, accessible online at https://jbi.global/critical-appraisal-tools, comprises 9 items for descriptive cross-sectional studies, 8 for analytical cross-sectional studies, and 11 for cohort studies. Response options include 'yes,' 'no,' 'not applicable,' and 'unclear.' Additionally, the tool features an overall appraisal option for the final decision to include or exclude a paper. Two authors independently conducted assessments, resolving any discrepancies through discussion and involving a third author.

### Outcome measurement

The first outcome was the mortality rate in patients with abdominal injuries. It was determined as the proportion of patients who died after sustaining abdominal injuries in all reviewed studies, calculated against the total number of patients with abdominal injuries. The second outcome was predictors of mortality in patients with abdominal injuries which was measured by adjusted odds ratio. In our review, a predictor was defined as an independent variable or factor that had a significant association with mortality among patients with abdominal injuries. A variable was considered a predictor if it showed a statistically significant association (*p*-value < 0.05) with the outcome of mortality in the multivariable analysis. Alternatively, it met the criteria for predictor if the adjusted odds ratio (aOR) did not cross 1.

### Data extraction and analysis

The data extraction format was prepared by authors using Excel 2013 software. The format consisted of the author(s) name, publication year, country, region, study design, sampling technique, sample size, participant’s age group, mechanism of injury, description of included patients, injury pattern elucidating the proportion with associated extra-abdominal injuries and/or multiple organ injuries, injury severity as assessed by different severity assessment score, the percentage of the most affected organ, mortality rate, and the adjusted odds ratio with its 95% CI of selected predictors of mortality. After extraction, data were exported to STATA version 17 statistical software for meta-analysis. Pooled analysis was conducted using a random-effects model with the Dersimonian-Laird method [19]. Finally, the results were presented using texts, tables, and different plots. The level of heterogeneity among the studies was assessed using the I-squared statistic, with values of 25%, 50%, and 75% indicating low, moderate, and high heterogeneity, respectively [19, 20]. In response to the value of heterogeneity, we performed subgroup analyses by study region, study design, participants’ age group, and mechanism of injury. To examine publication bias, we utilized funnel plots and performed Begg's and Egger's regression tests for a more objective assessment [21]. Trim and fill analyses were also performed. Furthermore, sensitivity analysis was employed to assess the influence of individual studies on the overall estimation.

## Results

### Search results

**Fig. 1:**
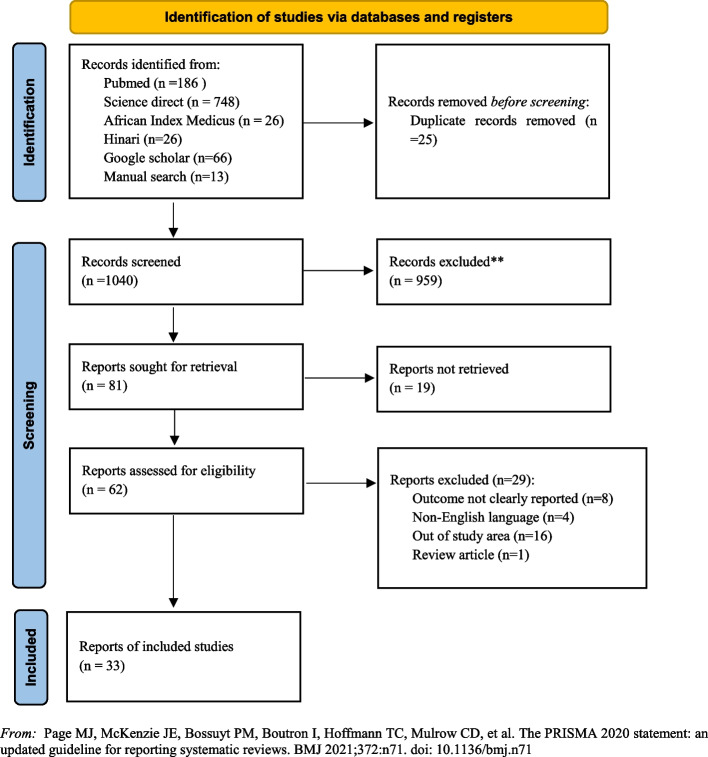
Flowchart of a selection of studies for a systematic review and meta-analysis of mortality and predictors in abdominal injury across sub-Saharan Africa, 2023

The initial search identified a total of 1,065 articles from various sources. After eliminating 25 duplicate articles, 1,040 unique articles remained. Subsequently, 959 articles that were considered irrelevant for this review were excluded, resulting in 81 articles selected for retrieval. Out of these, 19 articles lacked full-text availability and therefore could not be retrieved for further analysis. Following this, 62 full-text articles were thoroughly assessed based on the inclusion criteria. Among the assessed articles, 29 were excluded due to various reasons. Specifically, eight studies were excluded due to not reporting the outcome clearly [22–29], four were excluded as they were reported in a language other than English [30–33], sixteen were outside the predetermined study area [9, 34–48], and the remaining one was a review article [49]. Ultimately, 33 studies [15, 16, 50–80] met the inclusion criteria and were included in the meta-analysis (Fig. 1).

### Characteristics of reviewed studies

In this meta-analysis, there were thirty-three included studies published between 2000 and 2023, incorporating a cumulative sample size of 6,124 patients with abdominal injuries. All studies employed consecutive sampling methods. Among the included studies, 25 utilized a cross-sectional design, with eight opting for a cohort approach. Predominantly, the highest number (22 studies) were conducted in South Africa and Nigeria, evenly split with 11 studies in each country (Table 1).

**Table 1:** Descriptive summary of 33 studies included in the meta-analysis of mortality in abdominal injury across sub-Saharan Africa, 2023

**Author (s), Year**	**Country**	**Study Design**	**Sample size**	**Participants**	**Included patients**	**Mechanism of injury (%)**	**Injury pattern**	**Injury severity**	**Most affected organ (%)**	**Mortality (%)**
Abebe et al.,2019 [51]	Ethiopia	CS	129	Adults	Patients who underwent laparotomy for abdominal injury	All (38% blunt, 72% penetrating)	33.3% had EAI & 41.1% had multiple organ injury.	Not reported	SB (37.2)	8.5
Adejumo et al., 2015 [52]	Nigeria	CS	89	All	Patients presenting with abdominal trauma	All (37.1% blunt, 62.9% penetrating)	Not reported	Not reported	Spleen (29.8)	7.9
Adenuga et al., 2023 [53]	Nigeria	CS	87	Adults	Patients presenting with abdominal trauma	All (61% blunt, 39% penetrating)	11% had EAI	Not reported	SB (34.3)	17.2
Agbroko et al., 2019 [54]	Nigeria	CS	76	All	Patients presenting with abdominal trauma	All (40.8% blunt, 59.2% penetrating)	Not reported	Mean ISS was 15.8 ± 7.7	Not reported	7.8
Alli et al., 2005 [55]	Nigeria	CS	58	All	Patients presenting with abdominal trauma	Blunt only	Not reported	Not reported	Spleen (41.4)	17.2
Ameh et al., 2009 [56]	Nigeria	CS	82	Children	Patients presenting with abdominal trauma	All (69.5% blunt, 30.5% penetrating)	10.9% had EAI and 13.4% had multiple organ injury.	Not reported	Spleen (41.5)	13.8
Ayoade et al., 2006 [57]	Nigeria	CS	77	All	Patients who underwent laparotomy for abdominal injury	All (79.2% blunt, 20.8% penetrating)	54.5% had EAI	Not reported	Spleen (40.2%)	13.0
Chalya et al., 2013 [58]	Tanzania	CS	396	All	Patients presenting with abdominal trauma	All (77.8% blunt, 22.2% penetrating)	31.3% had EAI	KTS II ≤ 6 (in 28.3%)	Spleen (75.9%)	17.9
Demeke et al., 2022 [59]	Ethiopia	CS	165	All	Patients presenting with abdominal trauma	All (48.5% blunt, 51.5% penetrating)	15.3% had EAI	RTS ≤4 (in 0.6%)	Spleen (23%)	3.6
Dodiyi-Manuel et al., 2015 [60]	Nigeria	CS	45	All	Patients presenting with abdominal trauma	All (26.7% blunt, 73.3% penetrating)	13.3% had EAI	Not reported	SB (37.4%)	4.4
Dogo et at., 2000 [61]	Nigeria	CS	50	All	Patients presenting with abdominal trauma	All (54% blunt, 46% penetrating)	10% had EAI	Not reported	Spleen (64%)	10.0
Eaton et al., 2017 [62]	Malawi	CS	470	Adults	A subset of patients with abdominal trauma from a large study of trauma patients	All (not reported separately)	Not reported	Not reported	Not reported	7.5
Howes et al., 2012 [63]	South Africa	CS	65	All	All civilian patients who underwent laparotomy for abdominal injury	Blunt only	13.8% had multiple organ injury.	Not reported	Liver (13.8%)	26.0
Idriss et al., 2018 [64]	Mauritania	CS	100	All	Patients presenting with abdominal trauma	All (32% blunt, 68% penetrating)	20% had multiple organ injury.	Not reported	SB (16%)	2.0
Kong et al., 2019 [65]	South Africa	CS	301	All	Patients who underwent laparotomy for organ evisceration from abdominal stab wounds	Penetrating only	1% patients had combined eviscerations	Not reported	70% had eviscerated SB	2.0
Koto et al., 2015 [15]	South Africa	CS	114	All	Patients with PAT managed by diagnostic and therapeutic laparoscopy	Penetrating only	No associated injury reported	Not reported	Not reported	1.9
Krige et al., 2016 [16]	South Africa	RC	75	All	Surgically treated patients with combined duodenal and pancreatic injuries	Penetrating only	92% had other associated intra-abdominal injuries.	Median RTS =7 (range 3.5-7.1)	40% had a severe pancreatic injury	28.0
Manguni et al., 2012 [66]	South Africa	PC	416	All	Patients presenting with abdominal trauma	All (10% blunt, 90% penetrating)	24.3% had EAI	Not reported		12.2
Musau et al.,2006 [68]	South Africa	PC	80	All	Patients presenting with abdominal trauma	All (33.8% blunt, 66.2% penetrating)	35% had EAI	Not reported	SB (35.3%)	12.5
MONZON et al.,2006 [67]	South Africa	CS	89	All	Patients presenting with abdominal trauma	Penetrating only	18% had EAI	Not reported	SB (74.2%)	16.8
Ntundu et al. 2019 [69]	Tanzania	PC	136	All	Patients who underwent laparotomy for abdominal injury	All (72.8% blunt, 27.2% penetrating)	65.4% had EAI	NISS ≥25 (48.5%)	Spleen (26.5%)	13.2
Ogbuanya et al., 2023 [71]	Nigeria	PC	398	Adults	Patients presenting with abdominal injuries from Civilian Conflicts	All (9.8% blunt, 90.2% penetrating)	19.6% had EAI	Not reported	SB (20.3%)	11.6
Ohene-Yeboah et al., 2010 [72]	Ghana	Mixed	411	Adults	Patients presenting with abdominal trauma	Penetrating only	15.5% had EAI	Not reported	SB (23.2%)	4.4
Ojo et al., 2016 [73]	Nigeria	PC	109	All	Patients presenting with abdominal trauma from civil crises	All (9.2% blunt, 90.8% penetrating)	40.4% had EAI	Not reported	SB (63.3%)	10.1
Oosthuizen et al., 20118 [75]	South Africa	CS	257	Adults	Surgically treated patients who sustained a colonic injury secondary to penetrating abdominal trauma	Penetrating only	Not reported	Not reported	39.3% had concurrent SB injury	10.0
Reid et al., 2022 [76]	South Africa	CS	136	Children	A subset of surgically treated patients with abdominal trauma from a large study of trauma patients	All (57.1% blunt, 42.9% penetrating)	Not reported	The mean ISS score was 12.8±7.7	SB (31.4%)	5.1
Sander et al., 2022 [77]	South Africa	PC	805	All	Patients presenting with abdominal trauma	Penetrating only	40.9% had EAI	Median ISS of 13 (IQR 9–22)	SB (29.9%)	7.2
Sheshe et al., 2017 [78]	Nigeria	CS	46	All	Patients presenting with abdominal trauma	All (23.9% blunt, 76.1% penetrating)	24% had multiple organ injury	Not reported	SB (24%)	16.7
Merwe et al., 2023 [79]	South Africa	CS	205	Adults	Patients presenting with abdominal trauma, undergoing DCS, and having an open abdomen post-surgery	All (21% blunt, 79% penetrating)	6.7% had EAI	Not reported	Not reported	26.8
Abdulkadir et al., 2022 [50]	Ethiopia	CS	352	All	Patients who underwent laparotomy for abdominal injury	Penetrating only	20% had EAI and 38% had multiple organ injury.	Not reported	SB (55.1%)	3.4
Nyongole et al., 2013 [70]	Tanzania	CS	92	All	Patients who underwent laparotomy for abdominal injury	All (65.3% blunt, 34.7% penetrating)	37% had EAI	Not reported	Spleen (19.8%)	7.6
Omer et al., 2014 [74]	Sudan	CS	85	All	Patients presenting with abdominal trauma	Penetrating only	8.2% had EAI	Not reported	SB (39.1%)	4.7
Abraha et al., 2023 [80]	Ethiopia	CS	128	All	Patients who underwent laparotomy for abdominal injury	Blunt only	17.2% had EAI	Not reported	SB (57.8%)	5.5

*Abbreviations*: *AIS* Abdominal injury score, *CS* Cross-sectional, *IQR* Interquartile range, *ISS* Injury severity score, *KTS II* Kampala Trauma Score II, *LB* Large bowel, *NISS* New injury severity score, *PAT* Penetrating abdominal trauma, *PC* Prospective cohort, *RC* Retrospective cohort, *RTS* Revised Trauma Score, *SB* Small bowel

### Risk bias assessment

The 33 studies meeting the inclusion criteria underwent evaluation using the JBI critical appraisal checklist. Notably, none of these studies were excluded during the appraisal process, thereby warranting the inclusion of all 33 studies for the analysis in this review.

### Meta-analysis

#### Publication bias

**Fig. 2:**
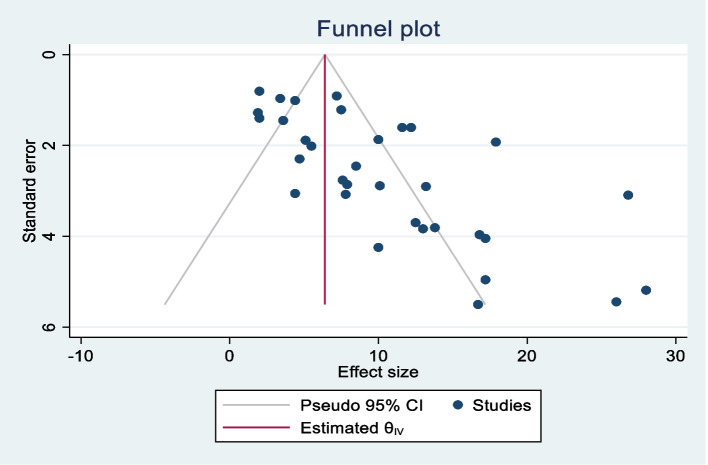
Funnel plot showing the asymmetric distribution of 33 articles on mortality in abdominal injury across sub-Saharan Africa, 2023

The funnel plot showed an asymmetric distribution (Fig. 2), while both Egger’s and Begg’s tests yielded statistically significant results (*p*<0.001) when estimating the mortality rate in abdominal injuries, suggesting the existence of publication bias. To assess its impact on the pooled analysis, trim fill analysis was conducted, resulting in the imputation of ten studies. Through this analysis, the pooled mortality rate for abdominal injuries became 6.73% (95% CI: 4.82%, 8.63%). As a result, the confidence interval indicates a minimal alteration in the overall effect size.

#### Sensitivity analysis

**Fig. 3:**
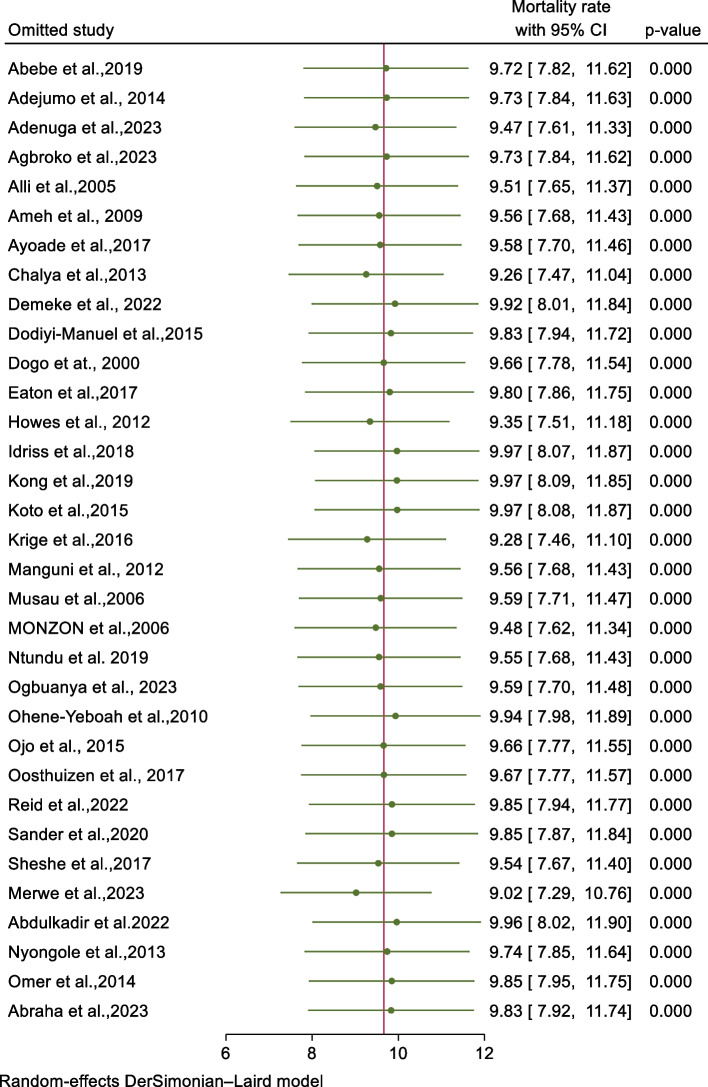
Sensitivity analysis of mortality in abdominal injury across sub-Saharan Africa, 2023 (*n*=33)

A random effect model result showed that no single study has influenced the overall pooled mortality rate in abdominal injuries across SSA (Fig. 3)

#### Mortality in abdominal injuries across sub-Sharan Africa

**Fig. 4:**
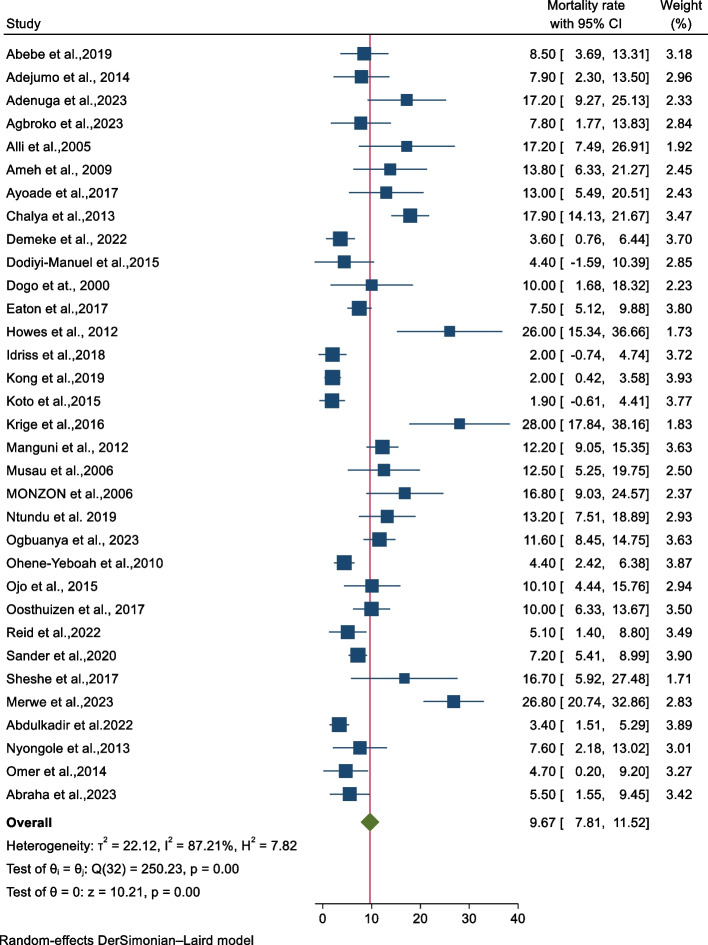
A forest plot for the pooled mortality rate of patients with abdominal injuries across sub-Saharan Africa, 2023 (*n*=33)

In the random effect model analysis, the overall mortality rate in abdominal injuries across SSA was 9.67% (95% CI; 7.81%, 11.52) with the heterogeneity index (*I*^*2*^ = 87.21%, *p* value< 0.001), showing substantial heterogeneity of different studies. In this analysis, the mortality rate for abdominal injury ranged from 1.9% [15] to 28 % [16]. The forest plot showed a distribution of weight across studies with a relatively narrow range, extending from 1.71% to 3.93% (Fig. 4).

#### Subgroup analysis of mortality in abdominal injuries across sub-Sharan Africa

Due to the observed heterogeneity, we conducted an exploration of potential factors associated with this variability, including region, study design, publication date, sample size, mechanism of injury, and participants’ age group using a meta-regression model. However, none of these factors showed a statistical significance.

In light of the substantial heterogeneity observed, we proceeded with a subgroup analysis based on region, study design, participants’ age group, and mechanism of injury. Particularly, in the Southern Africa region, the mortality rate for abdominal injuries was relatively higher at 11.41% (95% CI; 7.94, 14.89). Specifically, patients with blunt abdominal injuries demonstrated a significantly higher mortality rate of 15.51% (95% CI; 2.69, 28.32) (Table 2).

**Table 2:** Subgroup analysis of mortality rate in the current meta-analysis based on different variables

**Variables by category**	**No of studies**	**Pooled mortality rate (95% CI)**	***I***^***2***^** (*p*-value)**
Region			
East Africa	8	7.88 (4.20, 11.56)	87.50 (<0.001)
Southern Africa	12	11.41 (7.94, 14.89)	92.38 (<0.001)
West Africa	13	9.44 (6.58, 12.30)	75.67 (<0.001)
Study design			
Mixed	1	4.40 (2.42, 6.38)	-
Cohort	7	12.06 (8.81, 15.32)	76. 80 (<0.001)
Cross-sectional	25	9.25 (6.99, 11.50)	87.40 (<0.001)
Participant's age			
Adults	7	11.59 (7.39, 15.8)	90.16 (<0.001)
All age	24	9.15 (6.96, 11.35)	86.66 (<0.001)
Children	2	8.82 (0.38, 17.26)	76.13 (0.040)
Mechanism of injury			
All	21	10.51 (8.04, 12.97)	83.40 (<0.001)
Blunt	3	15.51 (2.69, 28.32)	87.11 (0.018)
Penetrating	9	6.44 (3.92, 8.97)	87.23 (<0.001)

#### Factors associated with mortality in abdominal injury across sub-Saharan Africa

Out of the articles we reviewed, five reported the role of shock at presentation in abdominal injury mortality [16, 51, 53, 58, 66]. In addition, different studies highlighted the significance of ICU admission [53, 66], blunt abdominal injury [51, 66], postoperative complications [58, 66], and damage control surgery (DCS) [16, 50] as predictors of mortality in abdominal injuries across SSA (Table 3).

**Table 3:** Summary of the predictors associated with mortality in abdominal injury across sub-Saharan Africa, 2023

**Factor**	**No of included studies**	**Pooled AOR (95% CI)**	**I**^**2**^** (*p*-value)**	**Reference category**
Shock at presentation	5	6.19 (3.70-10.38)	0.0(<0.001)	No
ICU admission	2	5.20 (2.38-11.38)	0.0(<0.001)	No
Blunt abdominal injury	2	8.18 (4.97-13.45)	0.0(<0.001)	Penetrating abdominal injury
Post-operative complication	2	8.17 (4.97-13.44)	0.0(<0.001)	No
Damage control surgery	2	4.62 (1.85-11.52)	0.0(0.001)	No

*Abbreviations*: *AOR* Adjusted odds ratio, *ICU* Intensive care unit

**Fig. 5:**
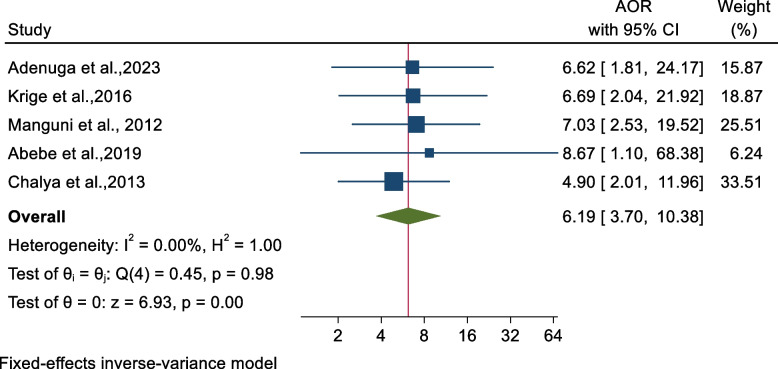
A forest plot showed the pooled effect of shock at admission on mortality in abdominal injury across sub-Saharan Africa, 2023

Consequently, the odds of mortality in patients with abdominal injuries were 6.19 times higher among patients presented with shock (AOR: 6. 19, 95% CI; 3.70-10.38) compared to those without shock (Fig. 5). Additionally, ICU admission (AOR: 5.20, 95% CI; 2.38-11.38), presence of postoperative complications (AOR: 8.17, 95% CI; 4.97-13.44), and the use of DCS (AOR: 4.62, 95% CI; 1.85-11.52) were associated with the higher odds of mortality. Moreover, the odds of mortality among patients with blunt abdominal injury were 8 times (AOR: 8.18, 95% CI; 4.97-13.45) compared with patients with penetrating abdominal injury (Table 3).

Different studies also reported additional predictors associated with mortality in abdominal injuries across SSA. These include delayed presentation, repeat surgery, advanced age, associated extra-abdominal injuries, and increased trauma severity scores (Table 4).

**Table 4:** Identified predictors of mortality in abdominal injuries across sub-Saharan Africa, 2023

**Author(s), Year, Ref**	**Publication year**	**Country**	**Study Design**	**Participants Age**	**Factors (AOR, 95%CI)**
Adenuga et al.,2023 [53]	2023	Nigeria	Cross-sectional	All	Late presentation >12 hours (5.6, 1.3-22.7), repeat surgery (6.2, 1.7-18.5)
Chalya et al., 2013 [58]	2013	Tanzania	Cross-sectional	All	Age>40yrs (3.5, 2.1-6.9), presence of associated extra-abdominal injuries (2.9, 1.1-8.3), Kampala Trauma Severity Score II < six (8.3, 3.4-9.9)
Krige et al., 2016 [16]	2016	South Africa	Retrospective cohort	All	Revised Trauma Score < 7.8 (6.2, 1.9-19.8), associated vascular injuries (4.2, 1.4-12.3)
Manguni et al., 2012 [66]	2012	South Africa	Prospective cohort	All	pH ≤7.2(4.82, 1.4-16.75), Injury severity score > 10 (3.04, 1.22-7.56), Age 41–50 years (8.11, 1.35-48.89), age ≥50 years (18.71, 3.62-96.64), four organs involved (11.49, 1.46-90.21)
Ntundu et al. 2019 [69]	2019	Tanzania	Prospective Cohort	All	New Injury Severity Score (15.5, 1.5-160)

## Discussion

This systematic review and meta-analysis aimed to assess the mortality rate and predictive factors in patients with abdominal injuries across sub-Saharan Africa. The pooled mortality rate for this region was determined to be 9.67% (95% CI; 7.81, 11.52). This finding is in line with findings observed in a prior systematic review [17] and a large-scale study [81]. In contrast, our findings demonstrate a lower mortality rate compared to a global review, which reported a 17% pooled mortality rate [14]. Discrepancies in inclusion criteria might contribute to the variations between the two reviews. In the current review, studies that reported mortality rates in all types of abdominal injuries were included. In contrast, the global review had a narrower focus, concentrating on patients who suffered hollow viscus injuries arising specifically from blunt abdominal trauma [14]. Indeed, the body of evidence consistently indicates that blunt abdominal injuries tend to escalate the risk of mortality [51, 66]. Our subgroup analysis also confirmed this, showing a higher proportion of death among studies conducted only in patients with blunt abdominal injuries. Contrarily, without an exact match for comparison, our findings demonstrate a higher result than what is observed in individual studies [82, 83].

This review also identified predictors of mortality in patients with abdominal injuries. Accordingly, the presence of shock upon presentation emerged as a significant predictor of mortality. In fact, shock reflects the state of physiological instability, indicating severe hemorrhage, directly impacting mortality rates [84]. This association implies the critical need for early recognition and immediate interventions to stabilize patients upon admission to reduce the risk of mortality.

In this review, significantly higher odds of mortality associated with blunt abdominal injuries were also observed. The possible rationale behind this association lies in the potential impediment to timely internal damage detection inherent in blunt injuries which ultimately causes a delay in employing a definitive management [85]. This delay, compounded with the complexity of recognizing concealed injuries, negatively affects the outcome. This emphasizes the need for tailored and specialized management strategies for patients presenting with blunt abdominal injuries to improve survival rates.

Our study also indicates that ICU admission after abdominal injury was associated with a higher mortality risk. This might be because patients admitted to the ICU are in critical conditions, which predisposes them to a higher likelihood of complications and mortality. Moreover, our analysis showed the link between post-operative complications and mortality in abdominal injuries. The result revealed that mortality was eight times higher among patients who had post-operative complications. This implies the importance of vigilant monitoring and comprehensive post-operative management to improve patient prognosis following surgeries for abdominal injury.

This review highlights a heightened mortality rate among patients with abdominal injuries subjected to damage control surgery (DCS). The plausible explanation for this association stems from the severity of underlying injuries that necessitate the implementation of damage control surgery. In cases of major abdominal trauma, DCS deviates from the immediate application of definitive surgery, opting instead for a cautious approach that avoids extensive procedures on unstable patients. DCS prioritizes addressing critical issues, such as rapid control of bleeding and contamination, during the initial operation. Subsequently, staged surgery is employed after achieving successful initial resuscitation [86]. However, the scarcity of intensive care units in many African settings, crucial for the effective restoration of physiological status, adversely impacts this approach and ultimately contributes to the observed elevated mortality associated with DCS. The link between DCS and increased mortality underscores the critical need for comprehensive trauma care strategies in regions where infrastructure limitations impact patient outcomes.

Although this review presents summarized evidence of mortality and its determinants in SSA, its scope was limited by excluding articles published in languages other than English as well as those without full texts. This exclusion limits the comprehensiveness of the review, potentially overlooking valuable findings from those studies. Furthermore, the application of consecutive sampling in all included studies, at the very least, might introduce bias associated with nonprobability sampling.

## Conclusion

The mortality rate in abdominal injuries across SSA was considerably high with substantial heterogeneity. The presence of shock upon presentation, ICU admission, blunt injury type, presence of postoperative complications, and the use of DCS were predictors of mortality. Addressing these predictors and implementing tailored strategies could significantly impact reducing mortality rates in patients with abdominal injury across the region.

### Supplementary Information

**Supplementary Material 1.** 

**Supplementary Material 2.** 

## Data Availability

All data supporting the findings of this study are available within the paper and its Supplementary Information.

## Abbreviations

DCS: Damage control surgery
ICU: Intensive care unit
SSA: Sub-Saharan Africa
