# Supplementary material for: Mortality and its predictors in abdominal injury across sub-Saharan Africa: systematic review and meta-analysis
Source: BMC Emerg Med. 2024 Apr 11;24:57. doi: 10.1186/s12873-024-00982-3 (PMC11008034; doi:10.1186/s12873-024-00982-3)
Supplement: Supplementary file 2 — Supplementary Material 2. [file 12873_2024_982_MOESM2_ESM.pdf]

Additional file 2: Search strategy

Concept 1: Mortality

Key terms used: Mortality, Death, Outcome

Mesh: "Mortality"[Mesh Terms]

Concept 2: predictors

Key terms used: Predictor, factor, associated factor

Mesh: "Epidemiologic Factors"[Mesh Terms]

Concept 3: Abdominal injury

Key terms used: Abdominal Trauma

Mesh: "Abdominal Injuries"[Mesh Terms]

Concept 4: Sub Saharan Africa

Mesh: "Africa South of the Sahara"[Mesh Terms]

Finally the search terms listed in the below table were used in different databases and search engine.

Data bases	Search terms	No identified
Pubmed	((("Mortality"[Mesh Terms] OR Death OR Outcome) OR ("Epidemiologic Factors"[Mesh Terms] OR Predictor OR factor OR "associated factor")) AND ((Abdominal Injuries[MeSH Terms]) OR (Abdominal Trauma))) AND (Africa South of the Sahara[MeSH Terms])	186
Cochrane Library	Mortality and its predictors in Abdominal injury	0
Science direct	(Mortality OR Death OR Outcome) AND (“Abdominal Injuries” OR “Abdominal Trauma”)	748
African Index Medicus	(Mortality OR Outcome) AND (Abdominal Injur* OR Abdominal trauma*)	26
Hinari	(TitleCombined:(Mortality)) AND (TitleCombined:(Abdominal Injuries))	26
Google scholar	allintitle: Abdominal injuries Mortality "Abdominal Injuries" OR "Abdominal Trauma" OR Mortality	66
Manual search		13
Total		1065
